# Soluble Factors from *Lactobacillus reuteri* CRL1098 Have Anti-Inflammatory Effects in Acute Lung Injury Induced by Lipopolysaccharide in Mice

**DOI:** 10.1371/journal.pone.0110027

**Published:** 2014-10-17

**Authors:** Milagros Griet, Hortensia Zelaya, Melina Valeria Mateos, Susana Salva, Guillermo Esteban Juarez, Graciela Font de Valdez, Julio Villena, Gabriela Alejandra Salvador, Ana Virginia Rodriguez

**Affiliations:** 1 Centro de Referencia para Lactobacilos (CERELA-CONICET), San Miguel de Tucumán, Tucumán, Argentina; 2 Instituto de Investigaciones Bioquímicas de Bahía Blanca (INIBIBB), Bahía Blanca, Buenos Aires, Argentina; University of Valencia, Spain

## Abstract

We have previously demonstrated that *Lactobacillus reuteri* CRL1098 soluble factors were able to reduce TNF-α production by human peripheral blood mononuclear cells. The aims of this study were to determine whether *L. reuteri* CRL1098 soluble factors were able to modulate *in vitro* the inflammatory response triggered by LPS in murine macrophages, to gain insight into the molecular mechanisms involved in the immunoregulatory effect, and to evaluate *in vivo* its capacity to exert anti-inflammatory actions in acute lung injury induced by LPS in mice. *In vitro* assays demonstrated that *L. reuteri* CRL1098 soluble factors significantly reduced the production of pro-inflammatory mediators (NO, COX-2, and Hsp70) and pro-inflammatory cytokines (TNF-α, and IL-6) caused by the stimulation of macrophages with LPS. NF-kB and PI3K inhibition by *L. reuteri* CRL1098 soluble factors contributed to these inhibitory effects. Inhibition of PI3K/Akt pathway and the diminished expression of CD14 could be involved in the immunoregulatory effect. In addition, our *in vivo* data proved that the LPS-induced secretion of the pro-inflammatory cytokines, inflammatory cells recruitment to the airways and inflammatory lung tissue damage were reduced in *L. reuteri* CRL1098 soluble factors treated mice, providing a new way to reduce excessive pulmonary inflammation.

## Introduction

The acute respiratory distress syndrome (ARDS) is a common disease that follows acute lung injury (ALI). ALI/ARDS is an important clinical syndrome of acute respiratory failure resulting from diffuse inflammation of the lung parenchyma, whose common clinical risk factors include sepsis and trauma. Pulmonary inflammation and pulmonary edema are two of the most important pathological findings in ALI/ARDS [1]. ALI/ARDS are characterized by neutrophil accumulation in the lung and apoptosis of the pulmonary epithelial cells, and leads to the destruction of the pulmonary epithelium and impairment of its barrier function [2], [3].

Lipopolysaccharide (LPS) has been referred to be an important risk factor of induced ALI and ARDS [1]–[4]. LPS is able to induce the inflammatory response by activating numerous inflammatory cells and result in acute lung injury. In fact, excessive inflammatory reactions and, in particular, neutrophil and macrophage activation have been implicated in ALI/ARDS pathogenesis [1]–[4]. Experimental administration of LPS, both systemically and intranasally, has been used to induce pulmonary inflammation in animal models of ALI. Administration of LPS in experimental animals causes the pathological condition of ongoing sepsis and concomitant ARDS-like lung injury, including neutrophil sequestration and lung edema [5].

In clinical cases, ALI/ARDS is a major problem that has a high mortality rate of 30–40% [4], [5]. Over the past decades, a variety of interventions and intensive care strategies have been used in treating patients with ALI/ARDS. However, only supportive treatments with lung protective ventilation and a fluid conservative strategy have been shown to be beneficial in improving morbidity and mortality [5]. Thus, novel therapeutic strategies are needed to improve the outcome of this disease.

Several studies have demonstrated that probiotic lactic acid bacteria (LAB) can exert their beneficial effect on the host through their immunomudulatory activity. Although most research concerning LAB-mediated immune modulation is focused on the gastrointestinal tract, recent studies have centered on whether these immunomudulatory probiotic LAB (immunobiotics) might beneficially modulate the respiratory immune system [6]. Research from the last decade demonstrates that immunobiotic LAB represent a promising resource for the modulation of respiratory immunity to improve protection against infectious and inflammatory respiratory diseases [7], [8].

Immunobiotics reported to exert their regulatory properties on cells of the immune system, including monocytes and macrophages, and to modulate the production of cytokines. A large number of studies have shown that the communication between immunobiotic LAB and host cells is multifactorial and involves a complex interaction between several receptors on the host cells that recognize multiple effector molecules on bacteria [9]. Moreover, most of these effector molecules have been found to be components of the cell-wall or associated to cell surface. In addition, during the last decade, a substantial body of scientific evidence has accumulated suggesting that certain extracellular components produced by probiotic LAB could be responsible for some of their mechanisms of action. The bacterial components that would be able to directly interact with the host mucosal cells include exopolysaccharides, lipoteichoic acids and surface-associated and extracellular proteins [10], [11]. In this regard, we demonstrated previously that *Lactobacillus reuteri* CRL1098 soluble factors are able to reduce tumor necrosis factor (TNF)-α production by human peripheral blood mononuclear cells (PBMC) [12], [13]. Considering the fact that numerous studies have shown that reducing the levels of TNF-α through the use of anti-TNF-α antibodies or soluble TNF receptors is a safe and efficacious treatment for inflammatory diseases, we hypothesized that *L. reuteri* CRL1098 soluble factors (LrS) could be used to modulate inflammation *in vivo*.

The aims of this study were to determine whether LrS were able to modulate *in vitro* the inflammatory response triggered by LPS in murine macrophages, to gain insight into the molecular mechanisms involved in the immunoregulatory effect, and to evaluate *in vivo* its capacity to exert anti-inflammatory actions in ALI induced by LPS in mice.

## Results

### LrS reduce the production of pro-inflammatory factors in LPS-challenged macrophages

**Figure 1:**
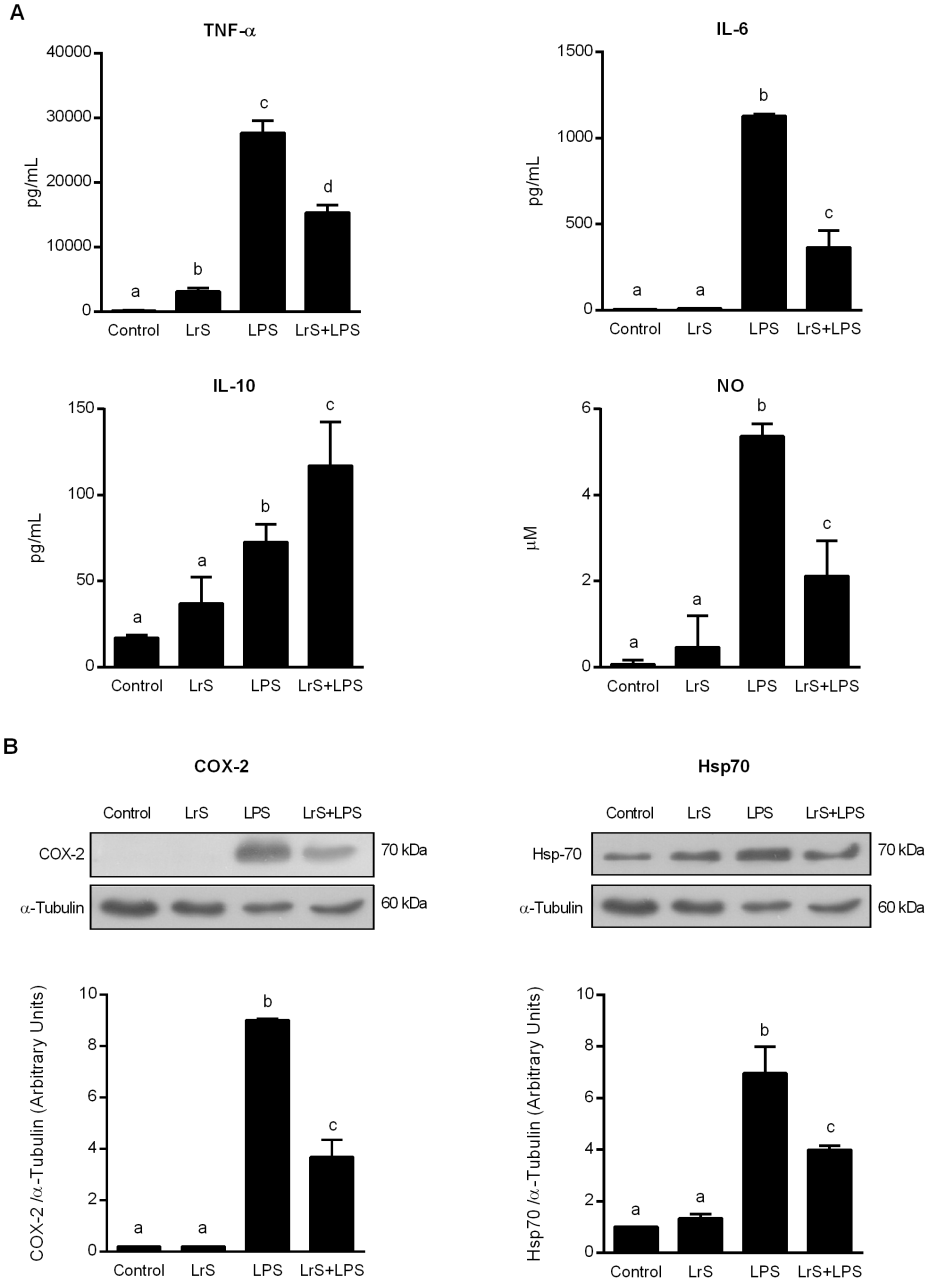
Effect of *Lactobacillus reuteri* CRL1098 soluble factors (LrS) on pro-inflammatory factors production by RAW 264.7 macrophages. RAW 264.7 cells were incubated in DMEM (control group) or DMEM with LrS (LrS group) for 4 hours. In a second set of experiments, control (LPS group)- and LrS (LrS+LPS group)-stimulated RAW 264.7 cells were challenged with LPS for 20 hours. A) Tumor necrosis factor (TNF)-α, interleukin (IL)-6, IL-10, and nitric oxide (NO) production were measured in culture supernatants. B) Cyclo-oxygenase (COX)-2 and heat shock protein (Hsp)-70 expression levels were evaluated by Western Blot analyses. Data shown is a representative result of three independent experiments. Values not sharing the same letter were significantly different (p value <0.05).

We first evaluated whether LrS were able to modulate the production of pro-inflammatory mediators in RAW 264.7 macrophages. As shown in Figure 1 treatment of macrophages with LrS significantly reduced TNF-α, but did not induce significant changes in the production of interleukin (IL)-6, IL-10 and nitric oxide (NO). No modifications in the expression of cyclo-oxygenase (COX)-2 or heat shock protein (Hsp)-70 were observed after LrS treatment. We also evaluated the effect of LrS in LPS-challenged RAW cells. Stimulation of cells with LPS significantly increased all the pro-inflammatory factors studied. However, the levels of IL-6, TNF-α, NO, COX-2, and Hsp-70 were significantly lower in LPS-challenged RAW cells treated with LrS (Figure 1). In addition, the levels of the immunoregulatory cytokine IL-10 were studied. Incubation of RAW cells with LPS augmented the production of IL-10; however the levels of that cytokine were significantly higher in LPS-challenged cells treated with LrS (Figure 1).

### LrS modulate NF-κB, Akt/PI3K and ERK ½ pathways in LPS-challenged macrophages

**Figure 2:**
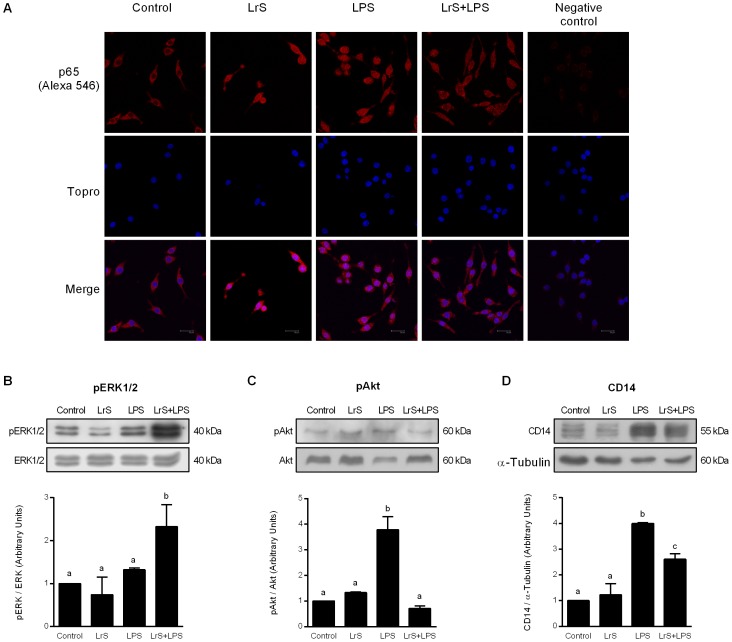
Effect of *Lactobacillus reuteri* CRL1098 soluble factors (LrS) on NF-κB, Akt, and ERK ½ pathways, and CD14 expression in RAW 264.7 macrophages. RAW 264.7 cells were incubated in DMEM (control group) or DMEM with LrS (LrS group) for 4 hours. In a second set of experiments, control (LPS group)- and LrS (LrS+LPS group)-stimulated RAW 264.7 cells were challenged with LPS for 20 hours. A) NF-κB pathway was evaluated by p65 translocation using immunofluorescence. B) Akt and ERK ½ phosphorylation, and CD14 expression were analyzed by Western Blot. Data shown is a representative result of three independent experiments. The bar graphs indicate the densitometry values of phosphorylated protein/non-phosphorylated protein expressed as ratio of control. Values not sharing the same letter were significantly different (p value <0.05).

In order to analyze the signaling pathways involved in the effect of LrS, RAW macrophages were incubated with LrS and, NF-κB, Akt, and ERK ½ pathways were evaluated by immunofluorescence or Western blot assays. As shown in Figure 2A, treatment with LPS or LrS increased the translocation of the p65 subunit of NF-κB from the cytosol to the nucleus of RAW macrophages indicating an activation of this pathway. Reduced translocation of the p65 subunit into the nucleus was observed in LPS-challenged RAW cells treated with LrS (Figure 2A). In addition, ERK ½ was activated by the coincubation of LPS with LrS. Challenge with LPS increased the phosphorylation of Akt indicating an activation of Akt/PI3K pathway (Figure 2C). In addition, we observed that levels of phosphorylated Akt were restored to control values in LPS-challenged RAW cells treated with LrS (Figure 2C).

We next investigated the expression of CD14 that is one important component of the receptor complex that detect LPS in macrophages. Treatment of RAW cells with LrS did not modify the expression of CD14 (Figure 2D). Stimulation of macrophages with LPS significantly increased the levels of CD14 protein, while LPS-challenged RAW cells treated with LrS showed significantly lower levels of CD14 (Figure 2D).

### LrS reduce apoptosis of LPS-challenged macrophages

**Figure 3:**
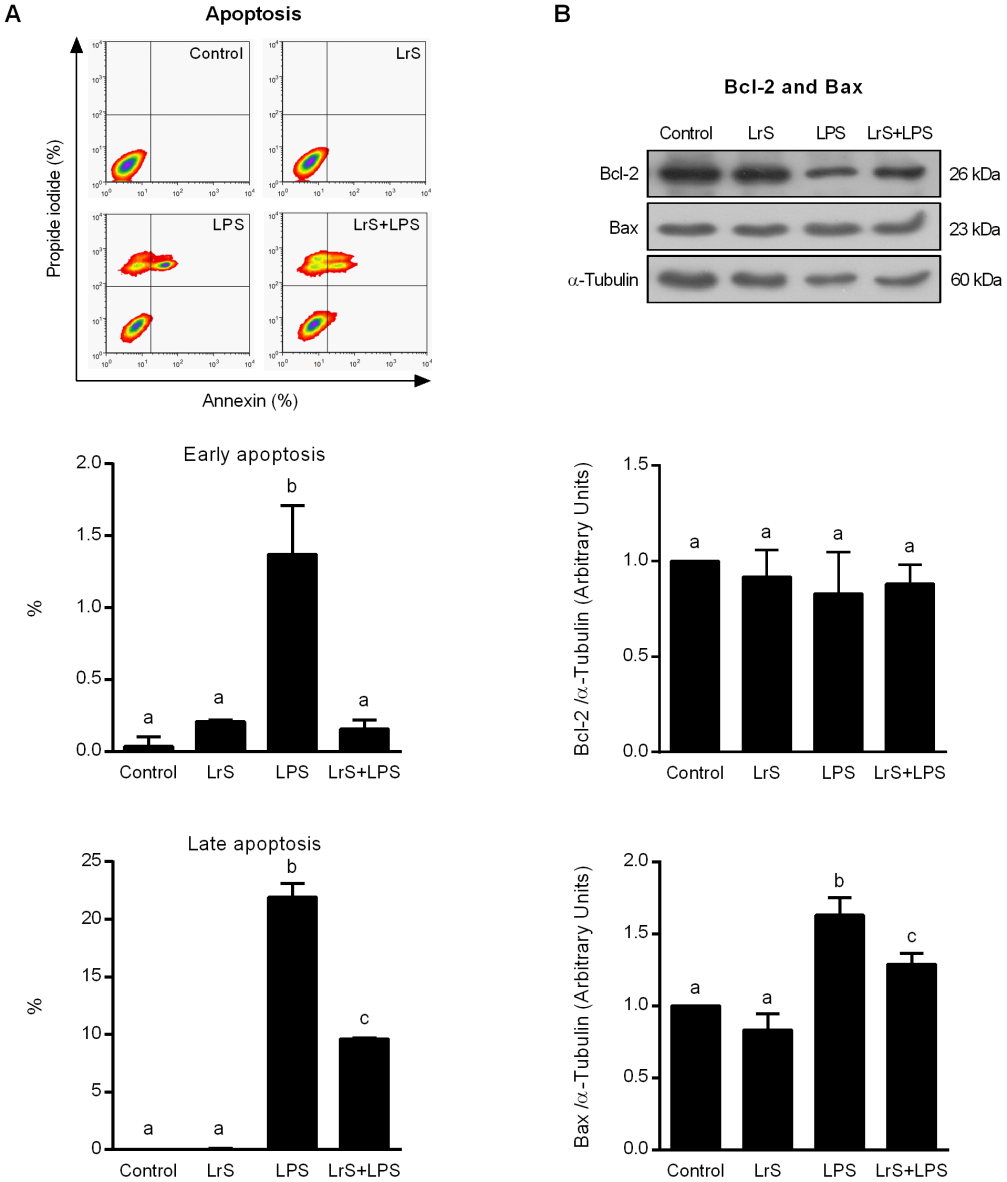
Effect of *Lactobacillus reuteri* CRL1098 soluble factors (LrS) on RAW 264.7 cells apoptosis. RAW 264.7 cells were incubated in DMEM (control group) or DMEM with LrS (LrS group) for 4 hours. In a second set of experiments, control (LPS group)- and LrS (LrS+LPS group)-stimulated RAW 264.7 cells were challenged with LPS for 20 hours. A) Apoptosis was evaluated by flow cytometry using Anexin V (AV) and propidium iodide (PI). AV^+^ and AV^+^PI^+^ macrophages were studied to evaluate early and late apoptosis respectively. B) Pro-apoptotic protein Bax, and anti-apoptotic factor Bcl-2 expressions were analyzed by Western Blot. Data shown is a representative result of three independent experiments. Values not sharing the same letter were significantly different (p value <0.05).

To evaluate apoptosis we used Anexin V (AV) and propidium iodide (PI) and determined the percentage of AV^+^ and AV^+^PI^+^ macrophages. As shown in Figure 3A, the percentage of both AV^+^ and AV^+^PI^+^ macrophages were significantly increased after the challenge with LPS, however LPS-challenged RAW cells treated with LrS showed values of AV^+^ and AV^+^PI^+^ cells that were lower than those observed in LPS controls. We also studied the levels of the pro-apoptotic protein Bax and anti-apoptotic factors Bcl-2 in macrophages. LPS challenge significantly increased levels of Bax in RAW cells while the levels of Bcl-2 were not modified (Figure 3B). LrS-treated cells showed levels of Bcl-2 that were not different from unchallenged control cells. Additionally, the levels of the pro-apoptotic protein Bax in LPS-challenged RAW cells treated with LrS were significantly lower than those observed in LPS controls (Figure 3B).

### Nasally administered LrS reduce lung injuries in LPS-challenged mice

**Figure 4:**
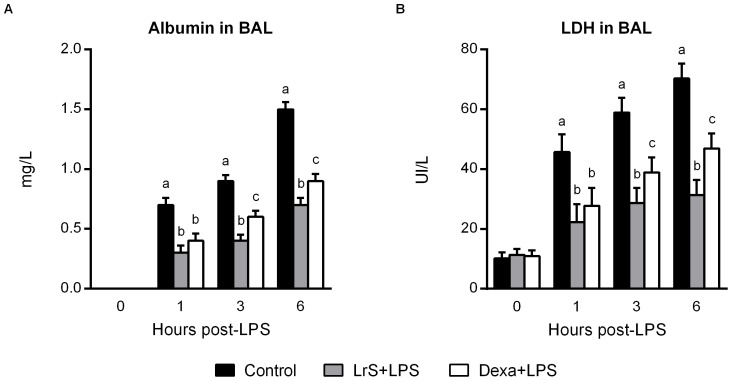
Effect of *Lactobacillus reuteri* CRL1098 soluble factors (LrS) on the lung tissue damage induced by LPS in mice. Male 6-week-old BALB/c mice were randomly allocated into three groups: control group, LrS-treated group, and dexamethasone group (Dexa+LPS). LrS or dexamethasone were given to animals by the nasal route immediately after nasal LPS administration. (A) Albumin content in bronchoalveolar lavages (BAL) was evaluated as a measure of increased permeability of the bronchoalveolar-capillarity barrier. (B) Lactate dehydrogenase (LDH) activity in BAL was evaluated as an indicator of general cytotoxicity. The results represent data from three independent experiments. Values not sharing the same letter were significantly different (p value <0.05).

We then evaluate whether LrS were able to exert anti-inflammatory activities *in vivo*. For this purpose we used a mice model of ALI induced by LPS administration. In these experiments mice were challenged intranasally with LPS with or without the co-administration of LrS. In addition, a third group of mice receiving LPS plus dexamethasone were used as controls for anti-inflammatory effect (Figure 4). Albumin content, a measure to quantitate increased permeability of the bronchoalveolar-capillarity barrier, and lactate dehydrogenase (LDH) activity, an indicator of general cytotoxicity, were determined in bronchoalveolar lavages (BAL). Both biochemical markers of lung injury were significantly increased after the challenge with LPS (Figure 4A and B). Treatment with dexamethasone or LrS significantly reduced the levels of albumin and LDH in BAL when compared to LPS controls. However, values of both biochemical markers were significantly lower in mice treated with LrS than those receiving dexamethasone (Figure 4A and B).

### Nasally administered LrS beneficially modulate inflammatory response in LPS-challenged mice

**Figure 5:**
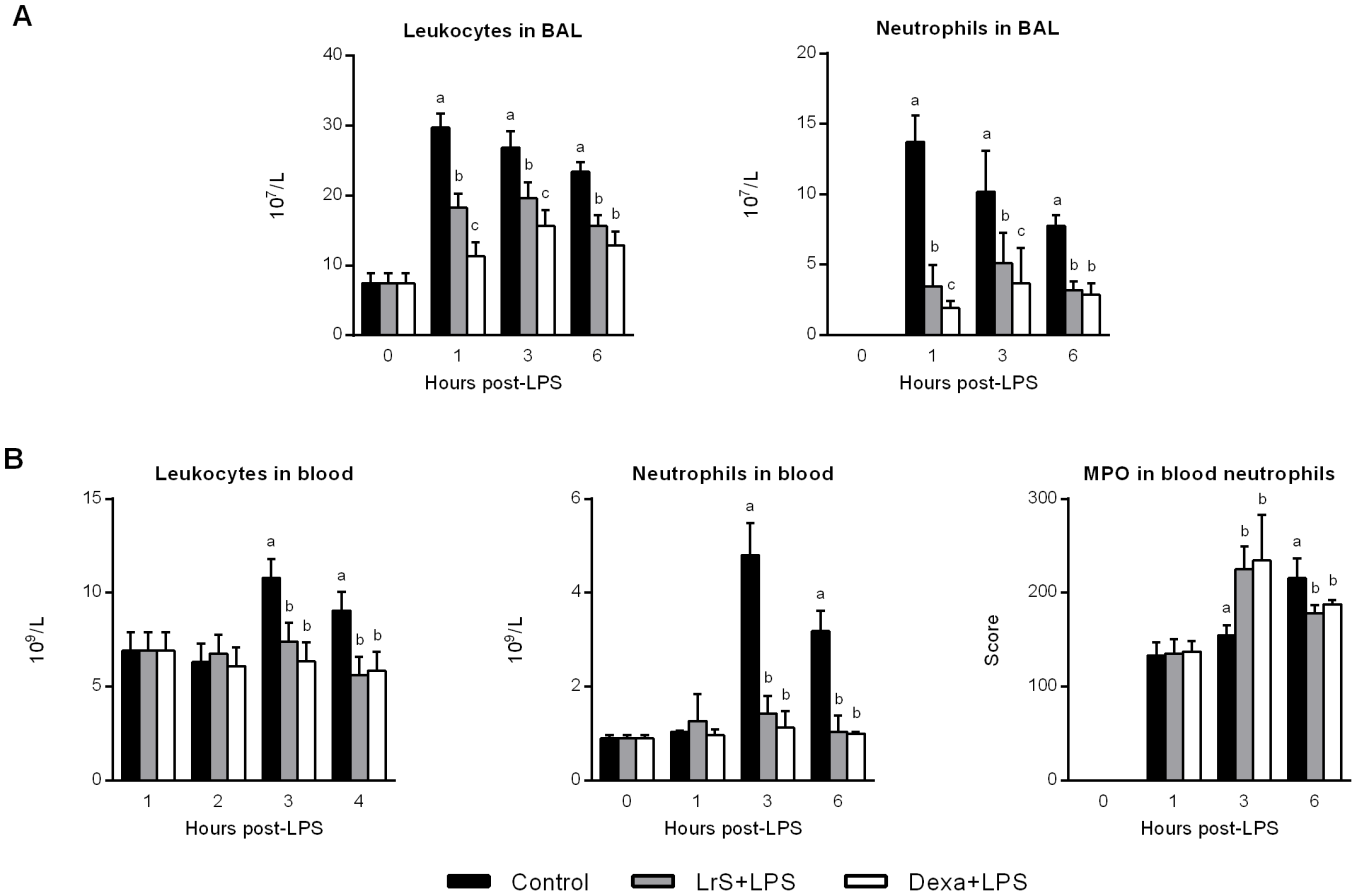
Effect of *Lactobacillus reuteri* CRL1098 soluble factors (LrS) on blood and respiratory leukocytes after nasal challenge with LPS. Male 6-week-old BALB/c mice were randomly allocated into three groups: control group, LrS-treated group, and dexamethasone group (Dexa+LPS). LrS or dexamethasone were given to animals by the nasal route immediately after nasal LPS administration. (A) Numbers of leucocytes and neutrophils in bronchoalveolar lavages (BAL). (B) Numbers of leucocytes and neutrophils, and myeloperoxidase (MPO) activity in blood. The results represent data from three independent experiments. Values not sharing the same letter were significantly different (p value <0.05).

**Figure 6:**
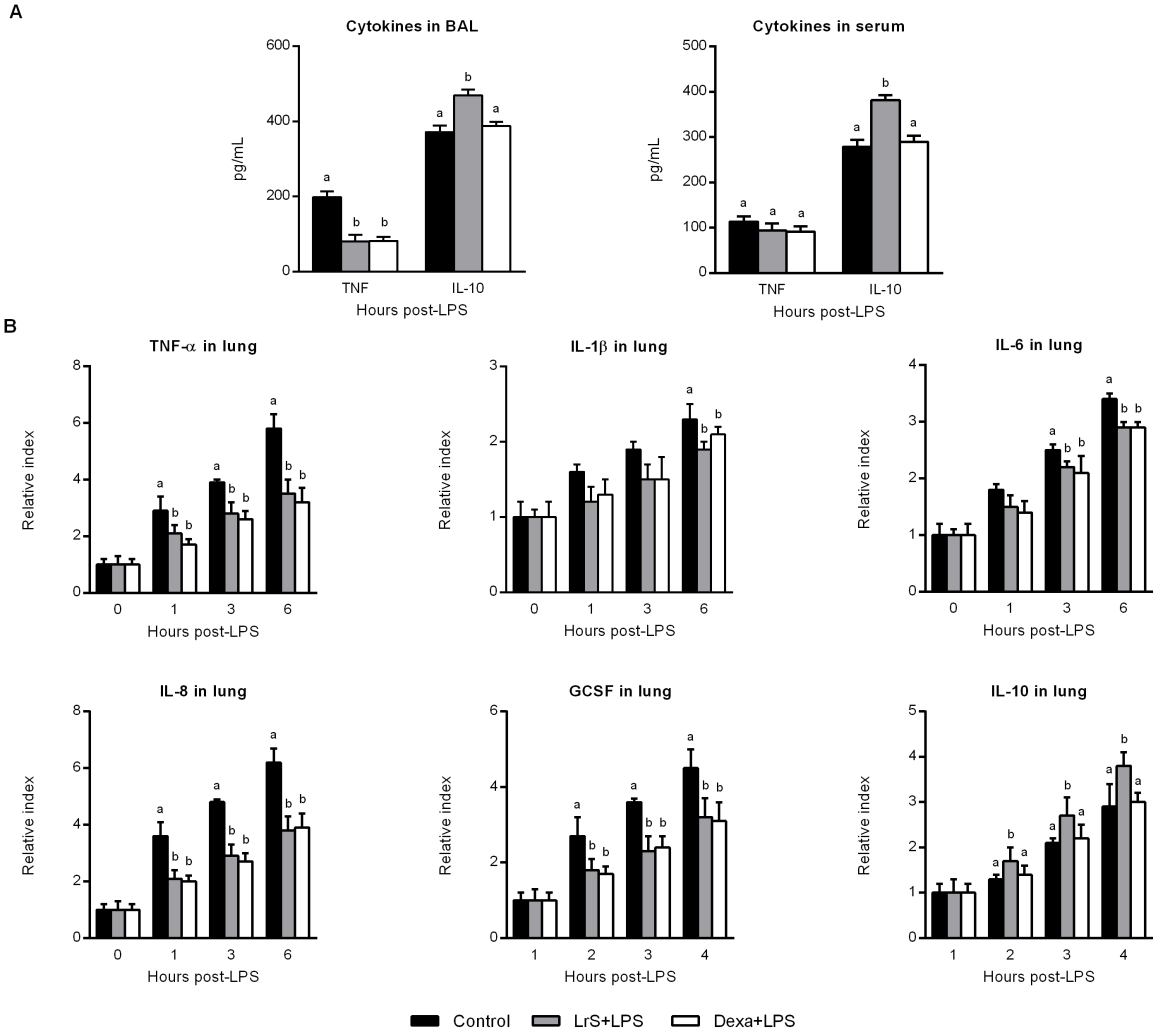
Effect of *Lactobacillus reuteri* CRL1098 soluble factors (LrS) on blood and respiratory cytokines after nasal challenge with LPS. Male 6-week-old BALB/c mice were randomly allocated into three groups: control group, LrS-treated group, and dexamethasone group (Dexa+LPS). LrS or dexamethasone were given to animals by the nasal route immediately after nasal LPS administration. (A) Blood and bronchoalveolar lavages (BAL) TNF-α, and IL-10 levels were determined by ELISA. (B) TNF-α, IL-1β, IL-6, IL-8, GCSF, and IL-10 expressions in lungs were determined by RT-PCR. The results represent data from three independent experiments. Values not sharing the same letter were significantly different (p value <0.05).

Challenge with LPS increased the number of blood and BAL leukocytes and neutrophils in all the experimental groups. Both dexamethasone and LrS significantly reduced the number of leukocytes and neutrophils in blood and in the respiratory tract when administered with LPS (Figure 5A and B). As shown in Figure 6A, stimulation with LPS induced significant increases in the levels of blood and BAL TNF-α, and IL-10. Both dexamethasone and LrS reduced the levels of BAL TNF-α when compared to LPS controls. Only mice receiving LrS showed values of blood and BAL IL-10 that were significantly higher than LPS or LPS+dexamethasone mice (Figure 6A). Finally, we determined the expression of TNF-α, IL-1β, IL-6, IL-8, GCSF, and IL-10 in lungs by RT-PCR. Stimulation with LPS significantly increased the expression levels of all the pro-inflammatory cytokines studied as well as IL-10 (Figure 6B). Both dexamethasone and LrS were able to significantly reduce the levels of lung TNF-α, IL-1β, IL-6, IL-8, and GCSF when compared to controls (Figure 6B). On the other hand, only LrS treatment was able to improve levels of IL-10 when compared to LPS controls (Figure 6B).

## Discussion

Inflammation is an essential aspect of the host’s response to invading pathogens to maintain a healthy state. However, aberrant inflammation leads to the up-regulation of several pro-inflammatory mediators including cytokines and enzymes. It was described that inducible nitric oxide synthase (iNOS) and COX-2 are important mediators of inflammation [14], [15]. Both iNOS and COX-2 are highly expressed in macrophages in response to inflammatory inducers like LPS, and are responsible for the production of NO and prostaglandin E2 (PGE2), respectively. Evidence indicates that the excessive release of NO by activated macrophages, and the activation of COX-2 with the increased conversion of arachidonic acid to PGE2; are associated with cytotoxicity at the site of inflammation, and have been correlated with the progression of inflammation-associated disorders [14], [15]. In addition, it was recently showed in mouse macrophages, that Hsp70 inhibition or knockdown prevented LPS-stimulated iNOS protein expression. Moreover, the preventing effect of Hsp70 inhibition on iNOS gene transcription was confirmed *in vivo* in endotoxemic mice [16]. The inflammatory response also activates macrophages to produce pro-inflammatory cytokines, such as IL-1β, IL-6, and TNF-α. The binding of TNF-α to surface receptors activates multiple signal transduction pathways and induces a secondary response by increasing the expression of several inflammatory cytokines that contribute to the biological activity of TNF-α [17]. Overproduction of TNF-α by macrophages has been also linked to several inflammatory diseases including cancer, sepsis, rheumatoid arthritis, and inflammatory bowel disease [17]. Thus, strategies to down-regulate the expression of these pro-inflammatory mediators during inflammatory processes are considered as potential therapeutic tools to alleviate the progression of inflammatory diseases caused by the activation of macrophages.

In the present study, we demonstrated that LrS reduced LPS-induced NO production, and Hsp70 and COX-2 expressions in RAW 264.7 macrophages. LrS also reduced LPS-induced production of the pro-inflammatory cytokines, TNF-α and IL-6. Furthermore, immunofluorescence results revealed that LrS reduced the translocation of the p65 subunit of NF-κB from the cytosol to the nucleus of RAW macrophages. NF-κB is required for maximal transcription and production of inflammatory mediators, and has been reported to play an important role in the pathogenesis of lung inflammatory diseases [18]. Since expression of pro-inflammatory mediators is tightly regulated at a transcriptional level via NF-κB pathway, the modulation of NF-κB pathway by LrS treatment could be a potential therapeutic application in inflammatory diseases.

Although the actual molecular mechanisms underlying the down-regulation of NF-κB activation and cytokine production by LrS remain unclear, our previous and present data indicate that LrS would exert its anti-inflammatory effect through the inhibition of Akt/PI3K pathway.

Some studies on signaling pathways that induce anti-inflammatory activity in RAW macrophages determined that the regulation of iNOS, COX-2, and pro-inflammatory cytokine production induced by LPS was mediated by the modulation of NF-κB through the inhibition of PI3K/Akt or MAPKs (ERK, JNK, and p38) pathways [19]. We demonstrated in this work an inhibition of NF-κB translocation to the nucleus in LrS-treated macrophages exposed to LPS as well as a reduction of Akt phosphorylation. In addition, we reported that LrS significantly increased the phosphorylation of ERK after the challenge with LPS, indicating that LrS does not exert its immunoregulatory effect through the inhibition of this MAPK. Morjaria *et al* [20] showed that imidazoquinoxalines are able to reduce TNF-α production in PBMC by activating p38 MAPK and inhibiting the PI3K/Akt pathway. In addition, it has been recently reported that polysaccharides found in medicinal plants are able to exert anti-inflammatory effects in RAW macrophages. Treatment of RAW cells with anti-inflammatory polysaccharides inhibited LPS-induced NF-κB and PI3K/Akt phosphorylation, while they had no effect on ERK, JNK, or p38 MAPKs [21]. Our present studies indicated that LrS is able to modulate NF-κB and to inhibit PI3K/Akt pathway, as described by other anti-inflammatory compounds [20], [21].

In addition, a number of molecules have been identified on macrophages that are involved in LPS responsiveness. These include TLR4, MD2 and CD14 [22]. Following its release from dividing or damaged bacteria, LPS is sequestered by an LPS-binding protein, which transports it to CD14 on the surface of macrophages. Subsequently LPS is transferred to MD2, a soluble protein that is associated with the extracellular domain of TLR4. Activation of TLR4 initiates cell signaling cascades leading to translocation of NF-κB into the nucleus and transcription of pro-inflammatory genes [22]. It was demonstrated that reduction in the expression of MD2 or CD14 significantly abolished the response of macrophages to LPS challenge. It was showed in alveolar macrophages that MD-2 siRNA reduces the transcription and protein expression of MD-2 efficiently and, that the LPS-induced secretion of the pro-inflammatory cytokines was reduced in MD-2 silenced cells [23]. In addition, Lei *et al.* [24] demonstrated that siRNA targeting CD14 inhibits TNF-α, MIP-2, and IL-6 secretion and NO production from LPS-induced RAW 264.7 cells. In line with these previous studies, we showed here that treatment of RAW macrophages with LrS significantly reduced the expression of CD14.

We also demonstrated that LrS is able to modify apoptosis of macrophages. Several studies showed that different lactobacilli strains may act as pro-apoptotic or anti-apoptotic effectors. For example, probiotic *Lactobacillus rhamnosus* produced molecules that promoted lymphocyte and THP-1 cells apoptosis [25]. In contrast, pre-treatment of rat hepatocytes with *Enterococcus lactis* IITRHR1 and *Lactobacillus acidophilus* MTCC447 inhibited the translocation of pro-apoptotic protein (Bax) and enhanced anti-apoptotic (Bcl-2) protein levels [26]. Our previous results demonstrated that LrS decreased apoptosis in PBMC to a half value compared to control cells [13]. The decreased apoptosis observed in PBMC and RAW macrophages treated with LrS can be the result of a combination of different pathways. Because of ERK ½ is associated to some anti-apoptotic pathways [27] and this kinase is activated in PBMC [13] and in RAW macrophages, the decreased apoptosis observed in those cells could be a consequence of the activation of ERK ½. In addition, the balance between anti-apoptotic and pro-apoptotic signal transduction pathways regulates the final response of cells to different stimuli. Our present data also suggest that LrS is able to modify the balance of pro-apoptotic Bax and anti-apoptotic Bcl-2 factors. In fact, LPS challenge significantly increased levels of Bax in macrophages while LrS-treated cells showed lower levels of this pro-apoptotic protein.

It has been showed that inhibition of pro-inflammatory cytokine production, cellular apoptosis, and NF-κB activation after LPS challenge is able to reduce lung injuries and improve survival rate [28], [29]. Therefore, we next aimed to evaluate *in vivo* the anti-inflammatory capacities of LrS by using a mice model of ALI.

In the absence of infection, alveolar macrophages prevent overreaction to inhaled antigen and maintain an immunologically suppressive environment. However, following antigenic challenge, macrophages can switch from protection into immunopathology. Alveolar macrophages play an important role in LPS-induced lung inflammation because of the early release of pro-inflammatory cytokines in response to LPS drives a cascade of inflammatory reactions. These activities can conduct to damage of lung architecture, influx of immune cells to the airways, and consequent impairment of gas exchange [30]. In ALI, pro-inflammatory cytokines that originate from alveolar macrophages recruit and activate neutrophils, which adhere to the affected capillary endothelium and migrate into air spaces, leading to severe lung injury [31]. Those changes were observed in our mice model of ALI. LPS administration significantly increased the number of BAL neutrophils and levels of lung TNF-α, IL-1β, IL-6, and IL-8. Moreover, LPS significantly increased the levels of BAL LDH and albumin indicating increased cytotoxicity and alterations of the alveolar barrier. We also observed that the nasal treatment with LrS was able to protect lungs from LPS-induced damage and that this effect was related to the capacity of LrS to significantly reduce the levels of TNF-α, IL-1β, IL-6, and IL-8 as well as the recruitment of inflammatory cells into the lungs. Considering the *in vitro* results in RAW cells, we can speculate that the treatment with LrS is able to functionally modulate respiratory macrophages to induce the anti-inflammatory effect. However, it should be consider that several other cells including epithelial cells and dendritic cells are also responsible of modulating immune responses in the respiratory tract [6], [7]. To evaluate *in vivo* the effect of LrS in those cell populations is an interesting topic for future research.

Surprisingly, the anti-inflammatory effects of LrS were comparable to the one achieved by dexamethasone. On the contrary, biomarkers of lung injury, albumin and LDH, were higher in dexamethasone-treated mice when compared to those receiving LrS. Therefore, the anti-inflammatory and protective effects of LrS are not only related to the reduction of pro-inflammatory factors but also to the increase of immunoregulatory cytokines as well. In fact, our results showed that LrS significantly augmented IL-10 levels in both LPS-challenged RAW cells and in BAL and serum of LPS-treated mice. It is known that an adequate balance of pro-inflammatory and anti-inflammatory factors is essential for limiting the detrimental effects of inflammation on the lung tissue. Several studies reported that IL-10 has an important protective role in controlling immunopathology during respiratory infections [32], [33] and ALI [34], [35]. Therefore, the induction of IL-10 by LrS could be an essential component of its protective effect in ALI.

## Conclusions

*In vitro* assays demonstrated that *L. reuteri* CRL1098 soluble factors significantly reduced the production of pro-inflammatory mediators (NO, COX-2, and Hsp70) and pro-inflammatory cytokines (TNF-α, and IL-6) caused by the stimulation of macrophages with LPS. NF-kB and PI3K inactivation by *L. reuteri* CRL1098 soluble factors contributes to these inhibitory effects. Inhibition of PI3K/Akt pathway and the diminished expression of CD14 could be involved in the immunoregulatory effect. In addition, our *in vivo* data proved that the LPS-induced secretion of the pro-inflammatory cytokines, inflammatory cells recruitment to the airways and inflammatory lung tissue damage were reduced in LrS treated mice, providing a new way to reduce strong pulmonary inflammation.

## Materials and Methods

### Bacteria cultures and supernatant obtaining

*Lactobacillus reuteri* CRL1098 (provided by the Culture Collection of Centro de Referencia para Lactobacilos, CERELA. Tucumán, Argentina) was grown in MRS (de Man, Rogosa and Sharpe medium, Britania, Buenos Aires, Argentina) up to mid logarithmic growth phase at 37°C (OD at 560 nm of approximately 0.50). The culture was centrifuged (8,000 *g* for 10 min), washed with phosphate-buffered saline (PBS) and RPMI 1640 medium with phenol red (GIBCO cat. N° 22400, Grand Island, NY, USA), resuspended in RPMI 1640 medium (GIBCO, Grand Island, NY, USA) and then propagated 4 h at 37°C, 5% CO_2_ in agitating conditions (4×10^7^ CFU/ml). The culture was centrifuged and cell-free supernatant was aseptically filtered using 0.22 µm pore size low protein binding cellulose acetate filters (Millipore, Bedford, MA, USA). The cell-free supernatant (pH 7.2) was used as recovered from the filtrates (non-diluted). The cell-free supernatant (pH 7.2) was frozen at −20°C for 24 h prior to the lyophilisation in a Lyovac GT2 lyophilizer (Leybold Cologne, Germany). Prior to the experiments, lyophilized cell-free supernatant was resuspended in medium.

### RAW 264.7cells and treatment with *L. reuteri* CRL1098 supernatant

Mouse macrophage cell line RAW 264.7 was cultured in DMEM supplemented with 10% fetal bovine serum (FBS), 100 IU/mL penicillin and 100 µg/mL streptomycin (DMEM-FBS) and maintained at 37°C in a 5% CO2 humidified incubator.

Cells were seeded in 35 mm-dishes or 24-well culture plates, lowed to adhere for 8 h at 37 °C in a humidified atmosphere of 5% CO2 prior to the addition of *Lactobacillus reuteri* supernatant (LrS) resuspended in medium (at a sub-cytotoxic dilution) and incubated for 4 h. Then, cultures were exposed to LPS from *E. coli* serotype O26: B6 (Sigma, St. Louis, MO, USA) at a final concentration of 15 µg/ml for 20 h. In all experiments, the cells were grown to 80–90% confluence and cell viability was assessed by the Trypan-blue assay (Sigma, St. Louis, MO, USA).

### Measurement of extracellular cytokine assay

Cytokines concentration was measured in cell free supernatants of RAW 264.7 cells using enzyme-linked immunosorbent assay kits (ELISA Ready-Set- Go!eBioscience, San Diego, CA, USA), including mouse TNF-α, IL-6, and IL-10.

### Measurement of NO in the culture media

The NO concentrations in supernatants of RAW 264.7 cells were determined by using Griess reagent (Promega Corporation, Madison, WI, USA). At prechosen intervals, 50 µl aliquots of medium were mixed with an equal volume of sulfanilamide solution (1% sulfanilamide in 5% phosphoric acid) and allowed to stand for 10 min at room temperature in the dark. Then, was added 50 µl of NED solution (0.1% N-1-naphthylenediamine dihydrochloride) and allowed to stand for 10 min at room temperature in the dark. The absorbance was measured at 540 nm with a microplate reader (VERSAmax, Molecular devices, Sunnyvale, CA, USA). Standard curve for sodium nitrite was used to acquire the level of NO produced by treated RAW264.7 cells.

### Western Blot Analyses

Treated RAW 264.7 cells were washed twice with 10mM PBS (pH 7.4) and then lysed with RIPA lysis buffer in the presence of protease inhibitors. Total protein concentrations were quantified using DC protein assay kit (Bio-Rad, Hercules, USA). Whole-cell lysates (30 µg) were resolved by 10% sodium dodecyl sulfate polyacrylamide gel electrophoresis (SDS-PAGE) and electrotransferred to polyvinylidene fluoride (PVDF) membranes (Santa Cruz Biotechnology, Santa Cruz, CA, USA) and probed with various primary antibodies. Membranes were blocked in 5% (w/v) albumin prior to incubation with the primary antibodies anti-COX-2, anti-Hsp70, anti-ERK ½, anti-p-ERK ½, anti-Akt, anti-p-Akt, anti-CD14, anti-Bcl-2, anti-Bax, and anti-α-Tubulin (Santa Cruz Biotechnology, Santa Cruz, CA, USA) and a peroxidase conjugated secondary antibody (anti-rabbit IgG or anti-mouse IgG). Blots were detected by chemiluminescence (Bio-Rad, Hercules, USA) using standard X-ray films (Santa Cruz Biotechnology, Santa Cruz, CA, USA). Intensity of individual bands was quantified using Image J densitometry software, and expressed relative to α-Tubulin signal, as a measure of protein relative abundance in the different conditions.

### Confocal Microscopy Analysis

RAW 264.7 cells were planted on the coverglass bottom dishes into 35 mm-dishesand then treated as described above. At prechosen intervals, RAW 264.7 cells were washed three times with PBS. The cells were fixed with 2% paraformaldehyde for 30 min, permeabilized with 0.1% Triton X-100 for 15 min, blocked with 2% BSA in PBS for 30 min, incubated with an anti-p65 primary antibody at room temperature for 1 h, sequentially incubated with an Alexa Fluor 546-conjugated secondary antibody at room temperature for 1 h in the dark, and finally incubated with TOPRO at room temperature for 5 min in the dark. Images were obtained using a Leica TCS SP2 AOBS confocal laser microscope.

### Flow cytometric analysis of apoptosis

Apoptosis was detected by flow cytometry using a FITC AV Apoptosis Detection Kit I (Becton Dickinson, San Jose, CA, USA). Briefly, RAW 264.7 cells were harvested by centrifugation, washed with cold PBS and resuspended in 1X Binding Buffer at a final concentration of 1×10^6^ cells/ml. Five micro liters AV and PI were added to 100 µl of the cell suspension (1×10^5^ cells) and incubated 15 min at room temperature in dark. 1X Binding Buffer (400 µl) was added to each sample before flow cytometry analysis. Unstained and AV or PI stained RAW 264.7 cells were used as controls of labeling. The percentage of viable and apoptotic cells was analyzed on a FACSCalibur flow cytometer (Becton Dickinson, San Jose, CA, USA) using the DeNovo software FCS Express V. 4 RUO. Viable cells were negatives to PI and AV. Early apoptotic cells were positives to AV and negatives to PI. Late apoptotic cells were positives to PI and AV.

### Animals

The experimental protocols were approved by the Ethical Committee of Animal Care from the Reference Centre for Lactobacilli (CERELA-CONICET, Tucuman, Argentina) under the protocol number LEF-2013-01 and all efforts were made to minimize suffering. Male 6-week-old BALB/c mice were obtained from the closed colony kept at CERELA (Chacabuco 145, San Miguel de Tucumán, Argentina). They were housed in plastic cages in a controlled atmosphere (22±2°C temperature, 55±2% humidity) with a 12 h light/dark cycle. ALI was induced in the mice by nasal administration of LPS from *E. coli* O55:B5 (8 mg/kg). The animals were randomly allocated into three groups: control group, LrS-treated group, and dexamethasone (2 mg/kg) group. LrS or dexamethasone was given to animals by nasal administration immediately after LPS administration. Samples were obtained at different times post LPS administration.

### Biochemical analyses in BAL

BAL samples were obtained according to the technique described previously [36]. Briefly the trachea was exposed and intubated with a catheter and 2 sequential lavages were performed in each mouse by injecting 0.5 mL of sterile PBS. The sample of fluid was centrifuged for 10 min at 900×g, the supernatant fluid was frozen at −70°C for subsequent biochemical analyses. Albumin content was determined to measure the increase of permeability of the bronchoalveolar-capillarity barrier was determined colorimetrically based on albumin binding to bromocresol green (Wiener Lab, Rosario, Argentina). The results were expressed as mg/L. LDH activity was determined as indicator of general cytotoxicity was determined by measuring the formation of a reduced form of nicotinamideadenine dinucleotide using Wiener Lab reagents and procedures (Wiener Lab, Rosario, Argentina). The results were expressed as U/L of BAL fluid [36].

### Total and differential blood leucocyte counts

Blood samples were obtained by cardiac puncture from sodium pentobarbital-anesthetized animals and were collected in tubes containing EDTA as an anticoagulant. Total number of leucocytes was determined with a hemocytometer. Differential cell counts were performed by counting 200 cells in blood smears stained with May Grünwald Giemsa stain using a light microscope (100x), and absolute cell numbers were calculated [36].

### Cytokine concentrations in serum and BAL

TNF-α, and IL-10 concentrations in serum and BAL, were measured with commercially available enzyme-linked immunosorbent assay (ELISA) technique kits following the manufacturer's recommendations (R&D Systems, MN, USA).

### Quantitative expression analysis by real-time PCR

We performed two-step real-time quantitative PCR to characterize the expression of IL-8, GM-CSF, IL-10, IL-1β, TNF-α and IL-6 mRNAs in lungs. Total RNA was isolated from each sample using TRIzol reagent (Invitrogen). All cDNAs were synthesized using a Quantitect reverse transcription (RT) kit (Qiagen, Tokyo, Japan) according to the manufacturer’s recommendations. Real-time quantitative PCR was carried out using a 7300 real-time PCR system (Applied Biosystems, Warrington, United Kingdom) and the Platinum SYBR green qPCR SuperMix uracil-DNA glycosylase (UDG) with 6-carboxyl-X-rhodamine (ROX) (Invitrogen). Primers were described previously [37], [38]. The PCR cycling conditions were 2 min at 50°C, followed by 2 min at 95°C, and then 40 cycles of 15 s at 95°C, 30 s at 60°C, and 30 s at 72°C. The reaction mixtures contained 5 ul of sample cDNA and 15 ul of master mix, which included the sense and antisense primers. Expression of β-actin was used to normalize cDNA levels for differences in total cDNA levels in the samples.

### Statistical analyses

All assays were performed at least in triplicate and the results were expressed as mean values with standard deviations. Statistical analyses were performed using GraphPad Prism 6 software (La Jolla, CA, USA). Comparisons were accomplished by ANOVA general linear model followed by Tukey's post-hoc test. Statistically significant differences were defined at a p value <0.05.
